# Development and Assessment of the Personal Emotional Capital Questionnaire for Adults

**DOI:** 10.3390/ijerph18041856

**Published:** 2021-02-14

**Authors:** Morteza Khazaei, Mark D. Holder, Fuschia M. Sirois, Lindsay G. Oades, Benedicte Gendron

**Affiliations:** 1 Ministry of Education, Abadan 6317984844, Iran; 2 Department of Psychology, University of British Columbia, Kelowna, BC V1V 1V7, Canada; mark.holder@ubc.ca; 3 Department of Psychology, University of Sheffield, Sheffield S1 2LT, UK; f.sirois@sheffield.ac.uk; 4 Centre for Positive Psychology, Melbourne Graduate School of Education, The University of Melbourne, Melbourne, VIC 3052, Australia; lindsay.oades@unimelb.edu.au; 5 Laboratoire Interdisciplinaire de Recherche en Didactique, Éducation et Formation, Paul Valéry University, 34000 Montpellier, France; benedicte.gendron@univ-montp3.fr

**Keywords:** personal emotional capital, mental health, depression, anxiety, stress

## Abstract

(1) Background: The present study developed and evaluated a personal emotional capital questionnaire (PECQ) for adults that assessed 10 domains of personal emotional capital. (2) Method: Initially, 100 items were created and then administered to students attending Semnan University and Semnan University of Medical Sciences in Iran. Of the 700 questionnaires distributed, 527 were completed in full. Students were sampledusing the multi-stage random cluster method. Exploratory factor analyses, Cronbach’s alpha, and test–retest reliability were used to evaluate the scale. (3) Results: The ten components ofthe PECQ were confirmed. Test–retest correlations after 30 days were high, as was Cronbach’s alpha (0.94). Thecomponents highly correlatedwith overall emotional capital. The PECQ displayed convergent validity as it positively correlated with the Keyes’s Mental Health Continuum—Short Form and students’GPAs. The PECQ displayed divergent validity as it negatively correlated with measures of depression, anxiety and stress (Depression Anxiety and Stress Scale (DASS21)). Differences in overall PECQ scores and its components were examined for several variables including gender, age, marital and employment status, academic program, and field of study. PECQ scores were not sensitive to the order of administering questionnaires. (4) Conclusion: The results suggest that the PECQ is a valid and reliable measure of personal emotional capital and supports its use in adults.

## 1. Introduction

Emotional capital refers to a set of resources that contribute to social cohesion, an individual’s professional development, organizational development, personal integrity, and personal, social and economic success [1]. High levels of emotional capital provide many advantages. Emotional capital is at the core of adaptation to changes in society and the workplace, and those with stronger emotional capital and higher levels of positive emotions can better communicate with others and enjoy greater job satisfaction, positive attitudes and performance [2,3,4]. Emotional capital consists of emotion-based knowledge, managerial skills, and abilities that assist in personal development and establishing group membership. It is a dynamic and structured resource that is shaped and re-shaped by socialization, activated in daily emotional activities, and can be considered a meaningful cultural activity that aids in attributing and interpreting events [5,6,7].

There are three type of emotional capital: personal, group and organizational [4]. All three are associated with a range of interpersonal advantages. For example, people with higher emotional capital report higher satisfaction with threatening, challenging and stressful relationships and are less reactive to their spouse’s negative behaviors and to daily tensions and threats to their relationships [8,9]. Mothers with low levels of emotional capital often experience low levels of social, cultural and economic capital, which affects their ability to care for and raise children. Emotional capital helps mothers maintain reliable social support [10,11,12,13,14]. Emotional capital plays a vital role in creating a sense of belonging for new immigrants [15,16]. Emotional capital assists in forming citizenship competencies that are required for human capital formation. Emotional capital is initially formed in the family, and helps people respond constructively instead of reacting hastily resulting in future regrets [17,18,19]. Developing these assets helps people become more competent and effective in their interactions with others in the workplace and community [17].

In addition to interpersonal relationships, high emotional capital is an asset in the workplace. Emotional capital is essential for people working in creative industries and businesses, where self-actualization is required, but there is little opportunity for secure employment [20]. Promoting emotional capital positively enhances the creativity and risk-taking that benefits entrepreneurship [21]. Strong emotional capital benefits the cognitive processes of managers’ decision-making [22,23,24,25]. Higher emotional capital provides individuals with greater capability to change their job, field of study and life circumstances [26]. High emotional capital benefits management knowledge, individuals, factories and institutions faced with strong competition in public, work and business environments [1]. The success of organizations largely depends on the emotional capital of their personnel [27,28].

Companies faced with struggles associated with downsizing, reduced funding and restructuring, need employees who possess high emotional capital in order to treat clients and the general public well. Companies struggle with preserving competitive advantages [4], and emotional capital assists in maintaining positive attitudes toward anxiety and stress [29]. Despite the rapid spread of knowledge between and within organizations, the success of organizations does not solely depend on employees’ ability to learn, but also on the ability of workers to cooperate and share knowledge. Thus, to be sustainable and successful, organizations must effectively develop and maintain the emotional capital of their employees [27]. Emotional capital is associated with superior sales of products with shorter production cycles [30].

Investing in emotional capital has better outcomes than investing in traditional capital. Considering the emotional capital of individuals during the hiring process and employment, and strengthening and maintaining employees’ emotional capital, benefits corporations and organizations [29]. Promoting managers’ emotional capital and improving their social performance enhances employee performance [31]. Maintaining high emotional capital benefits both business owners and job applicants, allowing companies to remain sustainable during external environmental changes and, providing customers with specific values [32,33]. 

Increased emotional capital is associated with benefits to medical patients, staff and institutions. For example, high emotional capital leads to greater tolerance of the complications and infections resulting from surgery. Job burnout and exhaustion experienced by doctors challenge health care systems. Social capital reduces job burnout and emotional capital plays a crucial role in improving relationships and increasing social capital. In healthcare, teamwork is critical and the need for emotional capital is essential for nurses [34,35,36,37,38,39,40].

Similar to healthcare, emotional capital is important in schools and universities. Effective leadership style in teaching requires high levels of emotional capital that teachers need to develop to meet the cognitive, emotional and social demands of their jobs [41]. Emotional capital improves teachers’ relationships with their colleagues and this leads to better collaborations in implementing school policies and approving laws in schools [42]. Teachers who work with autistic students are more likely to postpone leaving their job if they have higher emotional capital [43]. Emotional capital helps teachers establish a balance between individual development and professional skills and workplace well-being [44]. Improving personal emotional capital in students reduces dropout rates and increases academic achievement [1].

In summary, personal emotional capital is important in maintaining successful interpersonal relationships, enhancing citizenship skills, and attaining success in the workplace and schools. Investing in emotional capital benefits institutions, factories, treatment facilities and educational systems and families. Personal emotional capital can be learned and a first step for training programs is determining the initial level of emotional capital. To do this, researchers require a reliable and valid measure of emotional capital. However, researchers currently rely on emotional capital questionnaires with important limitations. For example, many of these questionnaires do not assess the full range of dimensions that comprise personal emotional capital [7,8]. Moreover, several measures are narrowly tailored to business and work environments [4,45,46], and thus are inappropriate to assess emotional capital in many domains. 

Therefore, we sought to develop a more comprehensive measure of personal emotional capital that was appropriate for use beyond a narrow range (only marital and work relations) of environments. Two researchers, Gendron and Newman, each provided different conceptualizations of emotional capital. However, their conceptualizations share 10 components: (1) Self-awareness (to recognize how one’s feelings and emotions affects personal opinions, attitudes and judgments); (2) Self-Confidence (to respect and like oneself and be confident in personal skills and abilities); (3) Self-Reliance (to take responsibility for oneself, back one’s own judgments and be self-reliant in developing and making significant decisions); (4) Straightforwardness (to give clear messages and express one’s feelings and points of view openly in a straightforward way and be comfortable challenging the views of others while demonstrating respect for their views); (5) Self-Actualization (to manage one’s reserves of emotional energy and maintain an effective level of work/life balance and thrive in setting challenging personal and professional goals); (6) Relationship Skills (to establish and maintain collaborative and rewarding relationships characterized by positive expectations); (7) Empathy (to understand the thoughts and feelings of others, and create resonant emotional connections with others); (8) Adaptability (to adapt one’s thinking, feelings and actions in response to changing circumstances, and be receptive to new ideas and tolerant of others); (9) Self-Control (to remain patient and manage one’s emotions well; restrain action and remain calm in stressful situations without losing control); and (10) Optimism (to sense opportunities, be resilient, and focus on the possibilities of what can be achieved even in the face of adversity [45,46,47,48]. 

The purpose of the present study was to develop and evaluate a personal emotional capital questionnaire (PECQ) for adults that assessed these 10 domains of personal emotional capital.

## 2. Materials and Methods

### 2.1. Participants

A sample of 700 students was selected from Semnan University (23 faculties and 16,500 students) and Semnan University of Medical Sciences (8 faculties and 3000 students) in Semnan, Iran through multi-stage random cluster sampling. Two criteria were considered for determining sample size using the PLS (Partial least squares path modeling) approach: ten times the maximum number of measurement model indices, and ten times the highest number of relationships in the structural sector [49]. The sample size required using the first criterion is 100, and using the second criterion is 100. Additionally, according to the Morgan sample size table and Kukran formula, 384 participants are sufficient for a society of 100,000. Based on any of these three guidelines, the current sample size of 700 participants is adequate. 

Participants were included if they indicated that they intended to complete the questionnaires after they were selected randomly and signed the consent form. Participants were excluded if they did not fully complete the questionnaires, ticked all the answers in only one column, or marked answers in a zigzag pattern.

Seven faculties were randomly selected from a list of faculties of the two universities, then 10 classes were selected randomly from each faculty (70 classes total), and 10 participants were randomly selected from each class of the selected classes. Some students declined to participate and they were replaced with students from their classes selected randomly. University students were used because they come from different regions of the country and they have different cultural, ethnic, religious, social class and family backgrounds. Additionally, they vary in age. Given their academic involvement, Iranian university students are more willing to complete questionnaires with greater commitment and accuracy.

The questionnaires were distributed among the selected students. Of the 700 questionnaires distributed, 527 were completed in full.

The mean and standard deviation of the participants’ age was 22.1 and 4.71, respectively, and ages ranged from 18 to 66 years. Table 1 shows the demographic characteristic of participants.

### 2.2. Procedure

Based on a review of the literature on emotional capital, 10 dimensions of emotional capital were identified and these components were defined based on the shared commonalities of Newman’s and Gendron’s theoretical conceptualizations. Based on the research literature and the definition of the dimensions, each dimension’s facets were identified including the aspects of life and behavior that comprised them. Using these facets and aspects, ten items were created to assess each dimension. Two items that assessed each dimension were reverse scored. The resulting 100 items were shown to fifty students who provided feedback on the clarity and appropriateness of them. Based on this feedback, items were revised. The revised items and the definitions of each component were given to 8 university professors who identified items that poorly matched the definitions. These identified items were revised and administered to 50 participants to further identify and revise inappropriate items. The final items were presented to eight university professors again, and they approved the questionnaire items and content. These final 100 items formed the emotional capital questionnaire and were administrated to 700 participants.

To evaluate the validity of the questionnaire, several additional scales (see Measures) were administered to assess mental health, depression, anxiety and stress. The GPA and demographic information of students were also recorded. Half of the participants completed the emotional capital questionnaire first, and the remaining half completed this questionnaire after the other scales were administered. 

After the participants were selected based on the steps described above (see Participants), and after they agreed to participate in the study and signed a consent form, their email addresses and mobile phone numbers were recorded. At this time, the participants were given the researcher’s email address and phone number along with a copy of the consent form. The participants were contacted via email or phone to determine the appropriate time for them to complete the questionnaires on campus. 

Of the 700 questionnaires distributed, 527 were completed in full. To help evaluate the reliability of the personal emotional capital questionnaire, 30 days after the initial administration, 100 students were randomly selected and the PECQ was re-administered.

The purpose of the research was explained to all participants. They were assured that their responses were confidential and their participation did not put them at risk. The research followed the guidelines for ethics prescribed by the American Psychological Association for working with human participants and Helsinki Declaration of 1964 and its later amendments.

### 2.3. Measures

*Adult’s Personal Emotional Capital Questionnaire (PECQ):* This questionnaire was designed to assess personal emotional capital in adults using a 4-point Likert scale: strongly agree = 4, agree = 3, disagree = 2, strongly disagree = 1, and in reverse-scored items, the scoring was strongly agree = 1, to strongly disagree = 4. By omitting 8 items based on a factor analysis, the questionnaire with 92 items displayed appropriate factor loads and Cronbach’s alpha was 0.94 (see Table 5). The PECQ questionnaire can be found in supplementary file.

*Mental Health Continuum Short version questionnaire (MHC-SF, Keyes, 2007)*: The MHC-SF is a short version of the MHC-LF. The MHC-SF consists of 14 items of which three evaluate emotional well-being, six evaluate the six dimensions of psychological well-being described by Ryff (1989), and five evaluate the five dimensions of social well-being described by Keyes (2007). The MHC-SF uses a Likert-type scale with 6 response options ranging from “never” (0) to “every day” (5) [50,51].

The MHC-SF assesses mental health, flourishing, and languishing according to Keyes’ mental health theory. This questionnaire is appropriate for adolescents and adults. A subjective well-being score is obtained from the total score of emotional well-being, psychological well-being and social well-being [51]. Cronbach’s alphas of the MHC-SF in the present study were 0.798, 0.91, 0.709 and 0.662 for the overall score, emotional well-being, psychological well-being and social well-being, respectively. Overall mean and SD were 37.59 and 10.25, respectively.

*Depression Anxiety and Stress Scale (DASS21, Lovibond and Lovibond, 1995)*: The DASS21 has three subscales each consisting of 7 items. The depression subscale includes items that specifically address depressive symptoms and correlate with aspects of sadness such as discomfort and worthlessness. The anxiety subscale correlates with signs of arousal, panic attacks, and fear (such as syncope and trembling). The stress subscale includes items that address stress, restlessness, and the tendency for hyperactivity in stressful activities. The DASS21 uses a four-option Likert-type scale ranging from “It never applies to me” (0) to “It is completely true of me” (3) [52,53]. The Cronbach’s alphas of this questionnaire in the present study were 0.875, 0.697, 0.728 and 0.758 for the overall score, Depression, Anxiety and Stress, respectively. Overall mean (SD) for stress, anxiety, and depression were 8.7 (4.2), 6.8 (3.9) and 7.6 (3.8), respectively.

### 2.4. Data Analysis

Confirmatory factor analyses were used to confirm the structure of the PECQ. The 92 remaining items with desirable factor loads confirmed the structure was comprised of ten components. Internal consistency and test–retest analysis after 30 days were also assessed for the PECQ. The MHC-SF scale and GPA, were used to evaluate the convergent validity of the PECQ. The DASS21 scale was used to evaluate the divergent validity of PECQ. A MANOVA (Multivariate analysis of variance) was used to compare the PECQ scores of male and female students. An independent t-test was used to compare PECQ scores from those who completed the questionnaire first to those who completed it last.

The confirmatory factor analysis in this research was done with Smart PLS software (Developed by SmartPLS GmbH, Germany, Hamburg). The PLS (partial least squares path modeling) method has two parts: (1) the measurement model and (2) the structural model. To evaluate the fit of the first part (fitting of measuring the models), three models are used: index reliability, convergent validity and discriminant validity. Index reliability is measured using three criteria: (1) Cronbach’s alpha, (2) composite reliability, and (3) factor load coefficients. Discriminant validity is used by cross loading factor method and Fornell and Larker method. In the second part analysis (the fit of the structural model) these criteria are used: Goodness of Fit (*GOF*) index, prediction power index *Q*^2^, *T* values, *R* Square or $R^{2}$ Criterion [54].

We chose Smart PLS because unlike the first-generation approach to structural equation modeling (SEM) that was based on the experimental reproduction of the covariance matrix (such as Lisrel), the second-generation approach focuses on maximizing the variance of dependent variables predicted by independent variables. An important benefit of the partial least squares (PLS) method is the evaluation of hierarchical models, which is used repeatedly in the PLS path analysis to conceptualize a hierarchical model of obvious variables (items). Thus, a higher-order hidden variable can be derived from all the obvious variables (items) of the lower order. PLS is not sensitive to sample size and normality of data. The PLS method, unlike the first generation, is a multivariate statistical method that is valuable even when the distribution of variables is unknown, the independent variables are correlated, and there are multiple independent and dependent variables [54,55,56].

## 3. Results

### 3.1. Descriptive Statistics

The means and standard deviations of the questionnaire and its components are presented in Table 2 along with the correlations of emotional capital for internal consistency and test–retest analysis of questionnaire after 30 days. 

### 3.2. Reliability of the PECQ

#### 3.2.1. Test–Retest Reliability and Internal Consistency

To verify the reliability of PECQ, the test was re-administered to 100 participants 30 days after the initial test. The test–retest correlations demonstrated acceptable reliability, 0.69 *< r* < 0.89, *ps* < 0.05. The correlation of components with the overall PECQ score was acceptable, 0.71 *< r* < 0.88, *ps* < 0.05 (see Table 2).

#### 3.2.2. Composite Reliability and Cronbach’s Alpha

Cronbach’s alpha reflects internal consistency. Values higher than 0.7 indicate acceptable reliability, although values above 0.6 are acceptable for variables with a small number of items [57,58,59]. The Cronbach’s alphas for all variables in the present study were above 0.6, which is considered acceptable given the small number of items. The Cronbach’s alpha for the PECQ in the present study was 0.94. 

Composite reliability values greater than 0.7, indicate good reliability, and values less than 0.6 indicate poor reliability [60]. As shown in Table 5, the coefficients of all latent variables are above 0.70, indicating acceptable levels. The composite reliability is a stronger criterion than the Cronbach’s alpha, which was introduced in the PLS method [61], and measures the adequacy of the items of a latent factor in its measurement. The Cronbach’s alpha gives an accurate indication of reliability when all indicators are loaded on a factor and all the indices in a model have the same weight. Because there were unequal correlations with different weights between indicators and factors in modeling the structural equation, the Cronbach’s alpha coefficient may not be reliable. Therefore, the composite reliability of factor loadings provides a more accurate estimate of reliability than the Cronbach’s alpha coefficient [62,63,64].

#### 3.2.3. Factor Loading and T Values of Items

The results of the confirmatory factor analysis and factor loadings of each item in relation to the purported structure are shown in Table 3.

T values above 1.96 indicate significance at the *p* < 0.05 level (95% confidence) and those above 3.23 indicate significance at the *p* < 0.001 level (99.9% confidence) [65].

The factor loads and significance coefficients were determined between the indices of each construct, indicating a significant relationship between items and components. The questionnaire had 100 items; based on the factor analysis, eight items were deleted due to poor factor loads ranging from 0.03 to 0.49. After factor analysis was used to identify desirable factor loads, 92 items remained.

### 3.3. Validity of the PECQ

#### 3.3.1. Convergent Validity

Fornell and Larcker [57] suggest that convergent validity requires coefficients to exceed 0.5. However, others consider a value of 0.4 as sufficient [58]. As shown in Table 5, all variables have coefficients of 0.4 or higher.

#### 3.3.2. Discriminant Validity

Discriminant validity was assessed with two methods: the cross loading factor method and Fornell–Larcker method.

##### Cross Loading Factor

Cross loading factor: The correlations between items and their constituents (see Table 4) were consistently greater than the correlations between those items and other factors indicating appropriate divergent validity [66].

##### The Fornell–Larker Method

*The Fornell–Larker method:* Test items need to show differentiation and separation from other items to demonstrate high discriminant validity [67]. This is shown when the square root of convergent validity of each component (the numbers on the diagonal of Table 5) is more than the maximum correlation of that component with other components. When this occurs, it indicates that each component is distinct and different from other components and does not measure a similar concept [67]. Using this approach, our results indicate that the PECQ shows good discriminant validity.

In summary, two different approaches both indicated that the PECQ has acceptable reliability and validity.

### 3.4. Qualitative Fitting of the Model and Structure Fit of the Model

#### 3.4.1. Power Index *Q^2^*

The quality of the structural model was assessed with the prediction power index *Q*^2^. Positive values indicate that the observed values are well restored, suggesting that the quality of the structural model is good [66]. Based on the *Q*^2^ Criterion of model, power prediction values 0.02, 0.15 and 0.35 are considered weak, moderate and strong, respectively [66]. Power prediction values ranged from 0.2343 to 0.3598 in Table 6.

#### 3.4.2. Goodness of Fit (*GOF*) Index

A Goodness of Fit (*GOF*) index considers both measuring and structural models and serves as a criterion for measuring the overall performance of the model [68]. Values of 0.01, 0.25 and 0.36 are considered as weak, moderate and strong, respectively. As shown in Table 6, the obtained *GOF* supports the overall desirability of the model.

#### 3.4.3. *T* Values

Using the structural model, the relationships between structures were evaluated. Considering the relationships between independent and dependent structures by using the relevant coefficient (*T* values), a significant analysis of the effects between research structures can be evaluated [69]. A Bootstrap test was used (with 500 sub-samples) for correcting the error. Construct level changes were used for calculating *T* values in the least-squares method to determine the significance of the path coefficients.

The most basic criterion for assessing the relationship between structures in the model (structural part) is the *T* value. Values greater than 1.96 indicate the correctness of the relationship between the structures and confirm the research hypothesis with a 95% confidence level. Of course, *T* values only show the correctness of the relations, not the magnitude of the relationship between the structures.

#### 3.4.4. *R* Square or *R*^2^ Criterion

*R Square or R*^2^*Criterion*: *R*^2^ is a criterion that is applied for connecting the measurement and structural parts of modeling of structural equations and demonstrates the effect that exogenous variables have on the endogenous variable. Therefore, this value is calculated only for the endogenous (dependent) structures of the model. Applying this criterion can reduce errors in the model of measurement and increase the variance between structures and indices controlled solely in PLS and are deprived in first-generation methods. Chin [70] categorized values of 0.19, 0.33 and 0.67 as weak, moderate and strong, respectively. Higher values indicate better fit of the model. The values of the determination coefficient displayed in Table 6 indicate a good fit.

**Figure 1:**
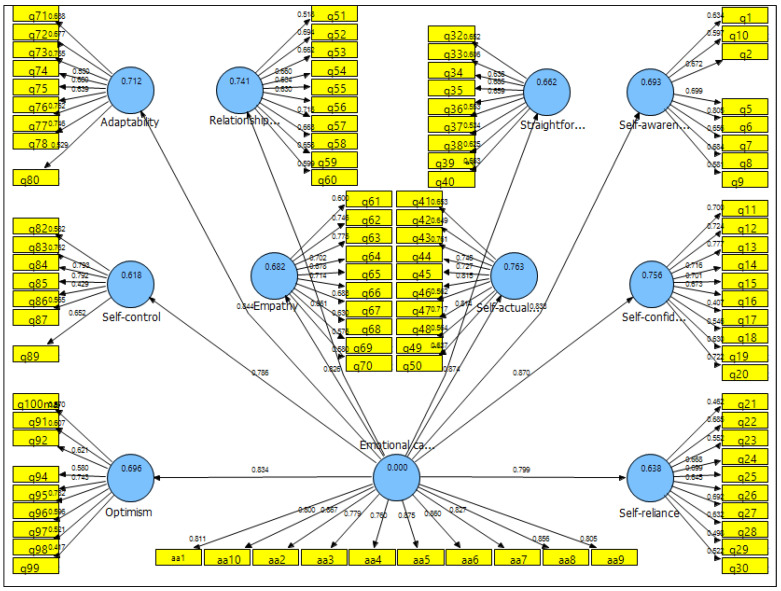
Factor loadings and $R^{2}$ coefficients of the structural model.

Figure 1 shows the factor load and coefficient of determination of the structural model and the coefficient of significance of the structural model.

### 3.5. Correlation of the Questionnaire with Convergent and Divergent Scales

As shown in Table 7, the overall PECQ score, as well as its ten components, were all significantly positively correlated with subjective well-being, emotional well-being, psychological well-being, social well-being and GPA. These correlations help demonstrate the convergent validity of the PECQ.

As shown in Table 7, the overall score of the personal emotional capital, as well as its ten components, were all significantly negatively correlated with measures of depression, anxiety, stress These correlations help demonstrate the divergent validity of the PECQ. 

### 3.6. Comparison of Participants’ Scores and Order Effect Evaluation

#### 3.6.1. Comparisons the Scores with MANOVA

PECQ scores were compared to individual characteristics (gender, marital status, age, employment status, academic program and field of study) using MANOVAs. Initially, in each stage, the presumptions of MANOVAs were tested using Leven’s and Box’s M tests prior to conducting each MANOVA. When considering gender, the Wilks’ lambda test was significant (*p* = 0.001, *F* = 5.728, *Wilks*’ *lambda* = 0.906), indicating that there is a difference between males and females in at least one variable. A MANOVA indicated that there were no significant gender differences for self-awareness, self-confidence, straightforwardness, relationship skills, self-control and optimism. However, females’ self-actualization (*p* < 0.05, *F* = 4.00), empathy (*p* = 0.01, *F* = 11.00), and adaptability scores (*p* < 0.05, *F* = 9.062) were significantly higher than males’ scores, and males’ self-reliance scores were higher than females’ scores (*p* = 0.000, *F* = 23.019). The Wilks’ lambda test was significant for marital status (*p* = 0.001, *F* = 3.158, *Wilks*’ *lambda* = 0.945) indicating a difference between married and single participants. A MANOVA indicated that self-reliance scores were higher for single students (*p* = 0.01, *F* = 18.666) and self-actualization scores were higher for married students (*p* = 0.01, *F* = 7.226). The Wilks’ lambda test was not significant for age (*p* = 0.393, *F* = 1.048, *Wilks*’ *lambda* = 0.908) or employment status (*p* = 0.400, *F* = 1.048, *Wilks*’ *lambda* = 0.981). When considering the academic program that students were enrolled in, the Wilks’ lambda test was significant (*p* = 0.001, *F* = 2.699, *Wilks*’ *lambda* = 0.866), indicating that emotional capital differed between the four academic programs in at least one variable. A MANOVA revealed a significant difference in three components: self-awareness scores were higher in Ph.D. students (*p* < 0.05, *F* = 3.233), self-control scores were higher in Ph.D. students (*p* = 0.01, *F* = 6.190), and adaptability scores were higher in M.A. students (*p* = 0.01, *F* = 7.717). When considering field of study, the Wilks’ lambda test was significant (*p* = 0.001, *F* = 3.190, *Wilks*’ *lambda* = 0.744), indicating that there is difference between five fields of study in at least one variable. A MANOVA indicated that the scores of students in medicine were higher for self-awareness (*p* < 0.05, *F* = 2.269), straightforwardness (*p* < 0.05, *F* = 2.862) and self-control (*p* = 0.01, *F* = 4.129). Scores of students in paramedicine were higher in empathy (*p* = 0.01, *F* = 3.253) and optimism (*p* < 0.05, *F* = 2.433). Scores of students in basic science were higher in self-confidence (*p* < 0.05, *F* = 2.599). Scores of engineering students were higher in self-reliance (*p* = 0.01, *F* = 5.670). Scores of students in humanities were higher in self-actualization (*p* < 0.05, *F* = 2.268). Scores of art students were higher in adaptability (*p* = 0.001, *F* = 5.831).

#### 3.6.2. Order Effect Evaluation

There was no order effect for the PECQ; the scores on the PECQ did not differ between those who took the test first or last, (*t* (98) = −0.692, *p* = 0. 491).

## 4. Discussion

The present research developed and then assessed the validity and reliability, of the PECQ, a new measure of personal emotional capital for adults. We confirmed that the PECQ has a 10-factor structure, and following the deletion of 8 items, the 92-item questionnaire displayed appropriate factor loadings and acceptable reliability, content validity, and discriminant validity. The PECQ was not sensitive to the order of taking the questionnaire but several components showed differences in emotional capital for gender, marital status, academic program, and field of study. The results demonstrate that the components are sensitive to individual differences and suggests that the components assess independent dimensions of emotional capital.

Current measures of emotional capital are limited because they assess only a few of the components of emotional capital with few items, and they are narrowly focused on specific environments and populations. One measure assesses only five components of emotional capital (self-awareness, self-regulation, motivation, self- awareness, and social skills) with 40 items [7]. Another measure assesses emotional capital with only six items and only considers emotional capital related to a spouse’s behavior by focusing only on marital relationships and married individuals [8]. 

The conceptualization of emotional capital varies across different fields. For example, management, sociology and psychology use different models and definitions of emotional capital. However, across fields, empirical research has consistently identified a host of benefits to individuals and institutions that are associated with high levels of emotional capital. Therefore, it may be desirable to develop programs that enhance emotional capital. To assess the efficacy of these programs, a comprehensive measure of emotional capital is required to determine the impact of these programs not only on overall emotional capital, but also on the components of emotional capital. The PECQ represents a more comprehensive measure because it assesses ten components of emotional capital. Given that the present research suggests that these components are distinct and independent from each other, researchers and health-care providers need to assess more than just overall emotional capital to more carefully identify which components are promoted through training interventions and which components are most amenable to change.

Though emotional capital has been assessed with questionnaires, an alternative approach that has been used in psychology, management and sociology, is the interview format. However, interviews only address overall emotional capital without considering the structure and components of emotional capital. The interview method is limited because to comprehensively assess the components of emotional capital in this format would require extensive time and, therefore, would be impractical for studies that require large samples. Furthermore, because interview formats are more vulnerable to interviewer bias than objective questionnaires, the results of different studies that rely on interviews may be less easily compared.

Though comprehensive measures of emotional capital have been developed, they are limited in that they are only suitable for narrowly defined populations and environments. For example, one measure developed by Newman assesses all ten dimensions of emotional capital; however, it is designed for middle and high-level managers and its items are exclusively tailored to target only work and business conditions [45,46]. 

Liu and Chen developed an emotional capital questionnaire with 12 indicators. Though comprehensive, this questionnaire assesses organizational, not personal emotional capital [4]. Therefore, this questionnaire does not address the need for a comprehensive measure of personal emotional capital. 

The PECQ may represent an improvement over current measures because it more comprehensively assesses emotional capital, and may be suitable for a wider range of people and environments than current measures. In developing the PECQ, attention was paid to creating items that would be appropriate for assessing many different groups and environments. However, we only administered the PECQ to university students in Iran. Additional studies are required to evaluate the appropriateness of this questionnaire for other groups in other locations before one can be confident of its wider suitability. Given that the PECQ has now been assessed with several criteria for validity and reliability using the PLS approach, this provides a level of confidence for its use.

The understanding of emotional capital adopted in psychology emphasizes two main benefits. First, high levels of emotional capital are associated with experiencing and sharing emotions that are more positive. Second, high levels of emotional capital are associated with better social relationships and the ability to maintain positive relationships even when they are challenged [9]. Among the various models of emotional capital, the competency-oriented model encompasses essential components for emotional capital and integrates emotional and social competencies, skills, and abilities in a conceptual network [45,46]. This model lends itself to guiding training programs designed to realize the benefits of enhancing emotional capital [45,46]. 

Among the competency-based models, Newman and Gendron’s models are the most well-known. In his model, Newman, guided by the three streams of emotional intelligence, identified twenty social and emotional competencies that contributed to personal and professional success. Based on the research literature and comparing the competencies, he extracted ten essential competencies for achieving personal and professional success and conceptualized them as emotional capital in a single network. Literature research suggests that people who demonstrate high levels of these ten competencies have a greater impact on others and are better able to build trust and better understand the needs of other people and motivate them [45,46]. The ten components that comprise Newman’s model can also be found in Gendron’s model of emotional capital though they have different labels. 

In this study, we aimed to develop a measure that comprehensively assessed the essential components of emotional capital and could be used for a wide variety of people in diverse environments based on the research literature. This measure could then be used to measure the development of emotional capital and assess changes in emotional capital arising from training programs designed to improve the essential components of emotional capital.

### Limitations and Implications

Though the present research confirmed the validity and reliability of a comprehensive measure of emotional capital, several limitations restrict the generalizability of these findings. The research sample was drawn from students attending two universities in Iran. The psychometrics of the questionnaire still need to be assessed in samples drawn from other universities, geographic regions, cultures, and ethnic, work and religious backgrounds. Additionally, the reliability, validity and sensitivity of the questionnaire should be assessed in specific populations including vulnerable populations (e.g., elderly and those with physical and mental health challenges). Additionally, the retest of the PECQ was conducted after a thirty-day interval. The stability of the scale would be more convincing if a longer interval was employed.

The PECQ was developed for and tested in adults. Future research could adapt the PECQ for other populations including adolescents. Moreover, the value of the questionnaire would be enhanced if future studies demonstrated the PECQ’s predictive validity. For example, it would be valuable to know if emotional capital scores measured during a job interview predicted future job evaluations and job success. Similarly, it would be of interest to determine if these scores measured during medical diagnoses or prior to clinical treatment or at the time of incarceration, predicted better outcomes. If the predictive validity of the questionnaire is confirmed, this may allow for interventions that are more effective. For example, if high levels of a specific profile of emotional capital components predicted greater marital satisfaction or improved outcomes in treating drug addiction, than interventions designed to increase these components could be utilized to increase success.

Perhaps specific components of emotional capital have greater predictive validity for different outcomes. This could possibly mean that only select portions of the questionnaire would need to be administered depending on the information required.

Future research could evaluate the relation of personal emotional capital and its components with personality traits. Perhaps certain personality traits predict personal emotional capital and its components. Determining these relations may be valuable to researchers who want to better understand personal emotional capital and develop ways to enhance it. For example, specific interventions designed to promote emotional capital may be more effective for a select group of domains of emotional capital or for individuals who possess certain personality traits. This information would help researchers appreciate the level of independence between the components of emotional capital. It would also aid researchers in developing effective interventions to increase the levels of emotional capital by identifying those components and individuals most amenable to change.

Furthermore, an Item Response Theory analysis of the individual items of the PECQ would be valuable. This analysis could confirm the value of each item and ensure that each item contributes to assessing personal emotional capital across the range for each component.

Additionally, the relationships between the PECQ and levels of family income and social class would be interesting to ascertain.

## 5. Conclusions

The present study reports on the development and assessment of the PECQ, a comprehensive measure of emotional capital that may be administered in a wide-range of environments. The PECQ demonstrated acceptable validity and reliability. The questionnaire may contribute to a better understanding of personal emotional capital and the development of ways to enhance it. Additionally, given that the PECQ assesses ten essential dimensions of emotional capital, it could help identify the specific domains that are amenable to change and enhanced by interventions. This information would help researchers appreciate the level of independence between the components of emotional capital. 

## Figures and Tables

**Table 1:** Demographic characteristics of students.

Gender		Age				Marital Status		Job Status		Academic Program			Field of Study					
Male	Female	18–20	20–30	30–40	40–66	Married	Single	Employed	Unemployed	PhD	M.A	B.A	Para medicine	Basic Science	Humanities	Art	Medicine	Engineering
46%	54%	19%	74%	5%	2%	17%	83%	19%	81%	5%	18%	77%	11%	13%	33%	11%	2%	30%

**Table 2:** Mean, *SD*, internal consistency and test–retest analysis of personal emotional capital questionnaire (PECQ) components.

Components	Number of Items	Mean of Men	*SD* Men	Mean of Women	*SD* Women	Mean	*SD*	Personal Emotional Capital	Re-Testing the Questionnaire
Self-awareness	8	25.08	4.07	25.04	4.11	25.07	4.09	0.83 **	0.887 **
Self-confidence	10	32.56	4.72	32.46	5.06	32.53	4.87	0.87 **	0.880 **
Self-reliance	10	31.85	4	30.5	4.03	31.23	4.06	0.71 **	0.888 **
Straightforwardness	9	29.14	4.52	28.59	4.12	28.89	4.34	0.81 **	0.874 **
Self-actualization	10	31.76	5.63	31.99	4.96	31.88	5.32	0.88 **	0.825 **
relationship skill	10	32.12	5.3	32.18	4.27	32.16	4.83	0.86 **	0.704 **
Empathy	10	32.24	5	32.82	4.76	32.52	4.88	0.83 **	0.693 **
Adaptability	9	25	4.2	25.48	4.14	25.22	4.17	0.81 **	0.745 **
Self-control	7	21.18	3.58	20.62	4.05	20.9	3.81	0.78 **	0.819 **
Optimism	9	27.91	4.34	27.32	4.36	27.65	4.36	0.82 **	0.778 **
Personal emotional capital	92	288.85	38.58	287.01	34.84	288.1	36.81	1	0.894 **

** *p* < 0.01.

**Table 3:** Factor loading and T values of items.

Component	Item	Factor Loading	*T* Values	Component	Items	Factor Loading	*T* Values
Self-awareness	q1	0.63	21.16	Relationship skills	q48	0.52	12.61
	q2	0.67	25.73		q49	0.69	24.39
	q3	0.70	27.75		q50	0.66	22.44
	q4	0.81	53.12		q51	0.66	22.22
	q5	0.66	21.18		q52	0.63	19.94
	q6	0.68	24.25		q53	0.63	19.95
	q7 *	0.58	17.42		q54	0.71	30.93
	q8 *	0.60	19.34		q55	0.66	23.84
Self- Confidence	q9	0.70	23.82		q56 *	0.66	20.27
	q10	0.72	34.24		q57 *	0.60	20.60
	q11	0.78	32.52	Empathy	q58	0.60	18.54
	q12	0.72	24.60		q59	0.75	29.50
	q13	0.70	27.72		q60	0.78	37.87
	q14	0.67	24.02		q61	0.70	28.76
	q15	0.41	9.39		q62	0.68	23.73
	q16	0.55	15.59		q63	0.71	26.05
	q17 *	0.63	21.58		q64	0.68	28.17
	q18 *	0.72	33.13		q65	0.63	20.86
Self-reliance	q19	0.46	10.62		q66 *	0.58	16.40
	q20	0.68	26.12		q67 *	0.58	15.21
	q21	0.55	15.36	Adaptability	q68	0.64	19.70
	q22	0.67	25.30		q69	0.68	25.44
	q23	0.70	24.69		q70	0.76	36.02
	q24	0.64	22.51		q71	0.53	9.78
	q25	0.69	26.53		q72	0.68	24.46
	q26	0.63	20.29		q73	0.64	24.88
	q27 *	0.50	12.21		q74	0.75	31.84
	q28 *	0.52	12.98		q75	0.75	30.70
Straightforwardness	q29	0.65	18.85		q76 *	0.53	13.86
	q30	0.61	16.80	Self-control	q77	0.58	14.28
	q31	0.64	21.26		q78	0.76	29.92
	q32	0.69	24.82		q79	0.79	40.34
	q33	0.66	20.79		q80	0.79	44.95
	q34	0.55	15.24		q81	0.43	10.06
	q35	0.53	14.61		q82	0.57	14.39
	q36 *	0.63	21.82		q83 *	0.65	21.27
	q37 *	0.66	20.48	Optimism	q84	0.61	17.62
Self-actualization	q38	0.65	24.52		q85	0.62	20.88
	q39	0.65	26.08		q86	0.58	14.83
	q40	0.76	32.68		q87	0.74	39.31
	q41	0.75	35.82		q88	0.73	31.31
	q42	0.73	31.58		q89	0.60	16.08
	q43	0.81	46.11		q90	0.52	13.29
	q44	0.56	15.66		q91 *	0.42	9.69
	q45	0.72	27.81		q92 *	0.57	16.56
	q46 *	0.56	14.02				
	q47 *	0.64	19.61				

* Reversed scored.

**Table 4:** Cross loading factor.

	Items	Self-Awareness	Self-Confidence	Self-Reliance	Straightforwardness	Self-Actualization	Relationship Skills	Empathy	Adaptability	Self-Control	Optimism
Components											
q1		0.63	0.48	0.34	0.32	0.39	0.39	0.35	0.44	0.40	0.36
q2		0.67	0.50	0.40	0.41	0.44	0.45	0.45	0.46	0.47	0.42
q3		0.70	0.58	0.45	0.43	0.57	0.49	0.44	0.47	0.45	0.48
q4		0.81	0.62	0.46	0.49	0.62	0.54	0.53	0.57	0.53	0.53
q5		0.66	0.50	0.38	0.35	0.46	0.36	0.36	0.40	0.41	0.42
q6		0.68	0.48	0.40	0.40	0.46	0.43	0.41	0.49	0.52	0.43
q7 *		0.58	0.43	0.44	0.44	0.49	0.45	0.38	0.40	0.40	0.48
q8 *		0.60	0.43	0.39	0.40	0.37	0.33	0.33	0.34	0.47	0.34
q9		0.51	0.70	0.46	0.47	0.47	0.46	0.43	0.43	0.39	0.44
q10		0.51	0.72	0.46	0.48	0.55	0.53	0.50	0.42	0.44	0.50
q11		0.55	0.78	0.40	0.45	0.54	0.49	0.43	0.47	0.46	0.52
q12		0.54	0.72	0.45	0.43	0.54	0.46	0.45	0.48	0.41	0.51
q13		0.61	0.70	0.58	0.52	0.59	0.55	0.54	0.58	0.50	0.59
q14		0.54	0.67	0.44	0.37	0.52	0.38	0.42	0.49	0.44	0.52
q15		0.38	0.41	0.28	0.32	0.27	0.26	0.21	0.30	0.29	0.26
q16		0.43	0.55	0.34	0.40	0.46	0.45	0.39	0.49	0.45	0.40
q17 *		0.42	0.63	0.53	0.50	0.47	0.45	0.39	0.38	0.30	0.43
q18 *		0.55	0.72	0.55	0.54	0.58	0.52	0.48	0.49	0.42	0.48
q19		0.30	0.27	0.46	0.32	0.25	0.25	0.28	0.22	0.32	0.23
q20		0.43	0.43	0.68	0.45	0.36	0.39	0.42	0.36	0.36	0.36
q21		0.25	0.32	0.55	0.32	0.24	0.29	0.28	0.20	0.21	0.24
q22		0.41	0.44	0.67	0.43	0.45	0.38	0.40	0.36	0.36	0.35
q23		0.41	0.51	0.70	0.52	0.48	0.48	0.45	0.44	0.43	0.46
q24		0.41	0.47	0.64	0.45	0.45	0.46	0.52	0.49	0.41	0.46
q25		0.46	0.53	0.69	0.49	0.55	0.49	0.46	0.51	0.51	0.47
q26		0.46	0.48	0.63	0.40	0.48	0.47	0.47	0.48	0.44	0.42
q27 *		0.25	0.30	0.50	0.40	0.24	0.31	0.26	0.21	0.15	0.26
q28 *		0.24	0.32	0.52	0.42	0.23	0.33	0.29	0.21	0.17	0.28
q29		0.40	0.45	0.43	0.65	0.39	0.43	0.40	0.38	0.33	0.34
q30		0.24	0.34	0.45	0.61	0.27	0.36	0.34	0.17	0.23	0.24
q31		0.27	0.37	0.46	0.64	0.38	0.40	0.41	0.32	0.32	0.36
q32		0.42	0.48	0.44	0.69	0.49	0.54	0.50	0.43	0.39	0.45
q33		0.45	0.44	0.49	0.66	0.49	0.55	0.52	0.51	0.41	0.51
q34		0.35	0.41	0.29	0.55	0.42	0.46	0.36	0.38	0.30	0.41
q35		0.29	0.29	0.31	0.53	0.31	0.33	0.36	0.26	0.24	0.20
q36 *		0.48	0.50	0.50	0.63	0.54	0.50	0.47	0.43	0.42	0.36
q37 *		0.44	0.49	0.48	0.66	0.44	0.48	0.38	0.36	0.40	0.37
q38		0.38	0.46	0.41	0.48	0.65	0.50	0.45	0.49	0.34	0.42
q39		0.41	0.43	0.45	0.51	0.65	0.50	0.47	0.42	0.33	0.43
q40		0.55	0.59	0.47	0.48	0.76	0.52	0.48	0.48	0.44	0.53
q41		0.55	0.60	0.49	0.49	0.75	0.51	0.53	0.54	0.49	0.51
q42		0.52	0.53	0.42	0.47	0.73	0.54	0.47	0.59	0.49	0.48
q43		0.58	0.59	0.49	0.55	0.81	0.56	0.59	0.60	0.53	0.55
q44		0.42	0.46	0.34	0.33	0.56	0.33	0.35	0.41	0.47	0.40
q45		0.55	0.56	0.47	0.46	0.72	0.50	0.53	0.52	0.51	0.53
q46 *		0.40	0.48	0.43	0.44	0.56	0.49	0.43	0.46	0.44	0.48
q47 *		0.45	0.51	0.41	0.42	0.64	0.46	0.44	0.43	0.41	0.48
q48		0.29	0.33	0.34	0.34	0.34	0.52	0.31	0.39	0.27	0.37
q49		0.49	0.52	0.48	0.48	0.51	0.69	0.50	0.52	0.42	0.53
q50		0.49	0.46	0.38	0.42	0.52	0.66	0.52	0.52	0.47	0.49
q51		0.47	0.42	0.45	0.48	0.45	0.66	0.51	0.45	0.44	0.45
q52		0.29	0.37	0.41	0.50	0.41	0.63	0.43	0.39	0.32	0.41
q53		0.40	0.38	0.30	0.47	0.48	0.63	0.43	0.46	0.37	0.43
q54		0.40	0.44	0.43	0.52	0.49	0.71	0.60	0.48	0.42	0.46
q55		0.47	0.50	0.45	0.44	0.44	0.66	0.56	0.46	0.44	0.46
q56 *		0.43	0.52	0.46	0.52	0.51	0.66	0.51	0.50	0.39	0.46
q57 *		0.41	0.48	0.43	0.52	0.45	0.60	0.43	0.35	0.33	0.41
q58		0.41	0.33	0.34	0.33	0.49	0.49	0.60	0.50	0.43	0.35
q59		0.45	0.46	0.43	0.45	0.51	0.52	0.75	0.47	0.46	0.42
q60		0.40	0.44	0.46	0.48	0.46	0.53	0.78	0.47	0.42	0.45
q61		0.33	0.40	0.41	0.45	0.38	0.47	0.70	0.31	0.32	0.39
q62		0.34	0.40	0.37	0.35	0.44	0.46	0.68	0.44	0.41	0.45
q63		0.44	0.46	0.47	0.46	0.46	0.49	0.71	0.47	0.41	0.43
q64		0.42	0.48	0.46	0.49	0.48	0.52	0.68	0.51	0.42	0.45
q65		0.48	0.47	0.46	0.49	0.54	0.52	0.63	0.53	0.48	0.45
q66 *		0.43	0.46	0.47	0.53	0.49	0.42	0.58	0.49	0.49	0.43
q67 *		0.38	0.41	0.44	0.45	0.37	0.49	0.58	0.35	0.32	0.41
q68		0.39	0.38	0.34	0.33	0.36	0.40	0.41	0.64	0.46	0.45
q69		0.46	0.43	0.46	0.38	0.45	0.51	0.51	0.68	0.53	0.50
q70		0.53	0.52	0.45	0.44	0.55	0.51	0.50	0.76	0.59	0.53
q71		0.27	0.32	0.23	0.24	0.37	0.33	0.29	0.53	0.28	0.34
q72		0.46	0.48	0.36	0.41	0.53	0.46	0.47	0.68	0.44	0.46
q73		0.38	0.44	0.46	0.45	0.43	0.50	0.50	0.64	0.41	0.55
q74		0.48	0.51	0.42	0.39	0.56	0.47	0.47	0.75	0.57	0.53
q75		0.53	0.51	0.42	0.43	0.58	0.54	0.51	0.75	0.53	0.56
q76*		0.38	0.37	0.41	0.41	0.36	0.45	0.37	0.53	0.39	0.36
q77		0.33	0.21	0.28	0.19	0.23	0.20	0.20	0.27	0.58	0.26
q78		0.49	0.47	0.39	0.39	0.45	0.42	0.42	0.53	0.76	0.52
q79		0.53	0.47	0.41	0.40	0.52	0.48	0.47	0.60	0.79	0.51
q80		0.56	0.46	0.44	0.40	0.56	0.48	0.53	0.61	0.79	0.48
q81		0.26	0.24	0.20	0.24	0.30	0.31	0.40	0.29	0.43	0.32
q82		0.40	0.39	0.34	0.37	0.38	0.37	0.35	0.44	0.57	0.48
q83 *		0.50	0.51	0.51	0.48	0.47	0.46	0.47	0.53	0.65	0.51
q84		0.44	0.45	0.26	0.29	0.46	0.42	0.38	0.50	0.53	0.61
q85		0.38	0.42	0.43	0.40	0.41	0.42	0.44	0.42	0.45	0.62
q86		0.40	0.38	0.31	0.30	0.38	0.34	0.30	0.38	0.45	0.58
q87		0.56	0.58	0.50	0.49	0.62	0.57	0.53	0.58	0.53	0.74
q88		0.44	0.52	0.44	0.43	0.49	0.55	0.48	0.56	0.49	0.73
q89		0.29	0.36	0.29	0.28	0.32	0.35	0.32	0.40	0.32	0.60
q90		0.31	0.35	0.34	0.35	0.30	0.35	0.31	0.32	0.35	0.52
q91 *		0.31	0.30	0.25	0.26	0.27	0.26	0.23	0.23	0.19	0.42
q92 *		0.37	0.42	0.38	0.37	0.46	0.43	0.37	0.40	0.31	0.57

* Reversed scored. In each column, the gray cells show the items that belong to the component.

**Table 5:** Convergent validity coefficients, Cronbach’s Alphas, composite reliability, Fornell and Larker correlation matrix and convergent and discriminant validity.

Component	Cronbach Alpha	Composite Reliability	Convergent Validity	Self-Awareness	Self-Confidence	Self-Reliance	Straightforwardness	Self-Actualization	Relationship Skills	Empathy	Adaptability	Self-Control	Optimism
Self-awareness	0.82	0.86	**0.45**	**0.67**									
Self-confidence	0.86	0.88	**0.45**	0.56	**0.67**								
Self-reliance	0.81	0.85	**0.4**	0.50	0.58	**0.63**							
Straightforwardness	0.80	0.85	**0.4**	0.50	0.57	0.58	**0.63**						
Self-actualization	0.87	0.89	**0.47**	0.61	0.55	0.53	0.57	**0.69**					
relationship skills	0.84	0.88	**0.42**	0.53	0.58	0.53	0.52	0.51	**0.65**				
Empathy	0.86	0.89	**0.45**	0.49	0.54	0.54	0.56	0.59	0.64	**0.67**			
Adaptability	0.84	0.88	**0.44**	0.57	0.59	0.51	0.49	0.62	0.60	0.57	**0.66**		
Self-control	0.78	0.84	**0.44**	0.58	0.52	0.47	0.46	0.56	0.51	0.53	0.55	**0.66**	
Optimism	0.78	0.84	**0.41**	0.54	0.56	0.49	0.48	0.59	0.58	0.52	0.53	0.59	**0.64**

The convergent validity column and the diagonal of the table are bolded.

**Table 6:** Structural model quality indices and structural model fitting of emotional capital and components.

Model Path		Structural Model			Model Quality	
Second-Order	First-Order	*β*	*T*	*R Square*	*Q* ^2^	*GOF*
Emotional Capital	Self-Awareness	0.83	52.742	0.69	0.3078	0.53
	Self-Confidence	0.87	75.067	0.7565	0.3341	
	Self- Reliance	0.799	43.917	0.6381	0.2343	
	Straightforwardness	0.814	44.240	0.6622	0.2539	
	Self-Actualization	0.874	64.93	0.7633	0.3598	
	Relationship Skills	0.861	65.182	0.7406	0.3072	
	Empathy	0.826	47.75	0.6815	0.3008	
	Adaptability	0.844	62.850	0.712	0.3110	
	Self- Control	0.78	42.621	0.62	0.2646	
	Optimism	0.834	56.705	0.6958	0.2526	

**Table 7:** Correlation between PECQ and subjective well-being, social well-being, psychological well-being, GPA, depression, anxiety, and stress.

Components	Subjective Wellbeing	Emotional Wellbeing	Psychological Wellbeing	Social Wellbeing	GPA	Depression	Anxiety	Stress
Emotional capital	0.618 *	0.578 *	0.379 *	0.324 *	0.685 **	−0.386 **	−0.380 *	−0.367 *
Self-awareness	0.758 **	0.793 *	0.381 *	0.676 **	0.658 **	−0.323 *	−0.257 *	−0.283 *
Self-confidence	0.500 *	0.485 *	0.247 *	0.293 *	0.617 **	−0.234 *	−0.280 *	−0.270 *
Self-reliance	0.367 *	0.513 *	0.167 *	0.100 *	0.465 **	−0.317 **	−0.440 *	−0.293 *
Straightforwardness	0.632 *	0.601 *	0.460 **	0.236 *	0.575 **	−0.240 *	−0.280 *	−0.254 *
Self-actualization	0.480 *	0.638 *	0.248 *	0.299 *	0.688 **	−0.424 **	−0.463 *	−0.450 *
relationship skills	0.623 *	0.533 *	0.421 *	0.327 *	0.646 **	−0.418 **	−0.480 **	−0.472 **
Empathy	0.539 *	0.559 *	0.299 *	0.261 *	0.606 **	−0.341 *	−0.250 *	−0.336 *
Adaptability	0.636 *	0.572 *	0.382 *	0.365 *	0.651 **	−0.234 *	−0.409 *	−0.328 *
Self-control	0.651 *	0.673 *	0.166 *	0.564 *	0.616 **	−0.282 *	−0.245 *	−0.305 *
Optimism	0.584 *	0.629 *	0.198 *	0.411 *	0.595 **	−0.263 *	−0.276 *	−0.481 *

** *p* < 0.01 and * *p* < 0.05.

## Data Availability

Data not available due to legal restrictions. The Ministry of Education did not agree for the students’ data to be shared publicly, so supporting data are not available.

## Supplementary Materials

The following are available online at https://www.mdpi.com/1660-4601/18/4/1856/s1, Word file 1: New Questionnaire with components definitions, Word file 2: New Questionnaire.
