# Supplementary material for: Development and Assessment of the Personal Emotional Capital Questionnaire for Adults
Source: Int J Environ Res Public Health. 2021 Feb 14;18(4):1856. doi: 10.3390/ijerph18041856 (PMC7918152; doi:10.3390/ijerph18041856)
Supplement: Supplementary file 1 [file ijerph-18-01856-s001.zip › ijerph-1066509-IR-SI-supplementary/New Questionnaire with components definitions.docx]

**Personal Emotional Capital Questionnaire for Adults (PECQ)**

A. Self- awareness: recognize how one’s feelings and emotions impact one’s personal opinions, attitudes and judgments.

1. I learn from my mistakes and failures and from these I gain experience.
2. I know my weaknesses and strengths.
3. I am a highly-motivated person (if I decide to do something, I will do it).
4. I can correct my mistakes and help myself progress in my job or learning and other affairs.
5. I can set goals for myself.
6. When I am aware of my mistakes and weaknesses or try to analyze them, I do not feel bad.
7. I do not understand why I feel miserable (I do not know why I fail).**
8. When I get upset or angry, I do or say things that I will regret later.**

B. Self-Confidence: respect for and liking one’s self, and being confident in personal skills and abilities.

1. I am a valuable person.
2. I am satisfied with my face and body (my looks).
3. I am proud of and satisfied with myself.
4. I can be as successful as other people.
5. I usually successfully accomplish the work that assigned to me or that I voluntarily undertake.
6. I love myself as I am.
7. I can defend my rights without the help of others.
8. I always know what to say to others.
9. I feel humiliated (inferior to others and worthless).**
10. Sometimes I feel like a useless person.**

C. Self-Reliance: take responsibility for oneself, support one’s own judgments, and be self-reliant in developing and making significant decisions.

1. If I participate in election polls, my vote is not affected by my friends, colleagues or family. I decide to whom I vote for.
2. I listen to others' opinions and advice, but I'm not dependent on others to make decisions. I make the final decision.
3. I do what I think is right, no matter that others are against me, or what they say.
4. When choosing clothes or hairstyles, I do not care about what others are wearing or what the latest fashion is. I choose clothes or hairstyles that I see fit and I like.
5. I can say no to others (colleagues, friends, family, etc.) even if they oppose me or put pressure on me.
6. I accept responsibility for my mistakes and failures.
7. What others think about me is not important to me and does not affect my thoughts, ideas and plans.
8. I do not feel the need to change my behavior or the way I talk, to satisfy others and gain their approval.
9. I always agree with others’ opinions when it comes to movies, books, sports teams, and actors. I don’t have any strong personal opinions.**
10. When talking to others at home, university or work, I have no idea of myself and I accept whatever others say.**

D. Straightforwardness (assertiveness): give clear messages and express one’s feelings and points of view openly in a straightforward way, and be comfortable challenging the views of others while demonstrating respect for their views.

29. If I do not understand something or have a question in the classroom or workplace, I will ask the professor or the person in charge.

1. When I am with my friends, colleagues or family, I say my own opinion even if everyone is opposed to me.
2. If I do something wrong, I have the courage to apologize.
3. If someone (family members, professors, employers and colleagues, etc.) criticizes or says something unfair about me, I can talk to them and defend myself.
4. I let others comment, even if they don’t agree with me.
5. When I need help, I can tell others and ask for help.
6. If after a purchase I find out that the seller has given me a defective or poor quality item, I can return the product.
7. If others (friends, colleagues, family members, etc.) insist that I do something, I do it, even if I would rather do something else (it's hard for me to say no to others).**
8. When a discussion is over, I regret that I didn’t have enough courage to say my opinion. **

E. Self-Actualization: manage one’s reserves of emotional energy, maintain an effective level of work/life balance, and thrive in setting challenging personal and professional goals.

1. There is always a way to progress.
2. Every day I can learn something new.
3. I know what my goal is in life and what I am looking for.
4. I can perform my duties well.
5. I know what my talents are.
6. I try to discover and develop my talents.
7. My life is neat, organized and based on a plan.
8. I try every day to improve my skills and abilities.
9. I refrain from trying to solve complex problems, doing hard works and tackling crises. **
10. I feel exhausted and frustrated.**

F. Relationship Skills: establish and maintain collaborative and rewarding relationships characterized by positive expectations.

1. I can talk to groups, colleagues or friends and encourage them to do something.
2. I can guide groups, friends or colleagues by providing different examples.
3. I do not interrupt others when they talking and I understand them well.
4. If someone criticizes me, I do not get angry. I listen to them.
5. I can mingle with the students from other fields of study at my university or with other people at a workplace and make friends with them.
6. If there is a problem between myself and others, I can talk that problem over with them and solve it.
7. In order to have good relationships with others, I pay attention to their feelings and attitudes.
8. I have a close relationship with my friends.
9. I cannot find a topic to talk about with others. **
10. I'm aloof and quiet among my classmates, colleagues, family members or relatives.**

G. Empathy: understand other people’s thoughts and feelings, and create close emotional connections with others.

1. Before I criticize someone, I put myself in their place, and I think if I were them, what I would do.
2. I try to understand others' needs and help them.
3. When my friends, colleagues or classmates at university have a problem, I sympathize with them.
4. When I see someone being oppressed, I'd love to help them.
5. When I see an angry or upset person, I try to talk to them and calm him down.
6. When I become aware of my friends’, colleagues’ or classmates’ problems, I try to offer them different solutions.
7. I respect other people’s opinions.
8. When I see someone get hurt, I keep calm so I can help him.
9. Hearing an opposing opinion makes me feel angry. **
10. The problems and needs of others do not concern me. **

H. Adaptability: adapt one’s thinking, feelings and actions in response to changing circumstances and be receptive to new ideas and tolerant of others.

1. When faced with a problem or difficult situation, I first gather information about it and consult with others.
2. When I try to solve a problem or manage a crisis, I first consider all the available options and then I come up with the best solution.
3. I can change my old habits.
4. If I do not reach a goal, I can define new goals for myself.
5. I can manage and plan household chores, college work, and tasks that should be done at my workplace.
6. There are several solutions to each problem or crisis.
7. When I fail, it's not hard for me to start again.
8. It is not hard for me to make a change in life (form good, new habits).
9. When faced with a problem or difficult situation, it is hard for me to decide on the appropriate solution. **

I. Self-Control: remain patient and manage one’s emotions well. Restrain action and remain calm in stressful situations without losing control.

1. I can control my feelings (I do not cry, I do not scream and shout, etc.).
2. During failures, I remain hopeful and cool and I do not get disappointed.
3. Even if I am in a bad situation, I can think straight and I do not lose control.
4. I know how to keep calm under difficult conditions.
5. When I know that if I do or say something, it will make things worse, I keep silent and don’t do anything.
6. I have no problem concentrating while studying and learning.
7. It is difficult for me to quit bad habits.**

J. Optimism: sense opportunities, be resilient, and focus on the possibilities of what can be achieved even in the face of adversity.

1. Every morning I am happy and cheerful.
2. I accept “where there is a will, there is way”.
3. People are inherently good and can be trusted.
4. I know that under the most difficult circumstances I can work to reach my goals.
5. Before I start a task, I feel that I can pull it off.
6. I always expect that problems and hardships will to lead to a happy ending. (of course this is not the case all the time).
7. I speak more about the future than the past.
8. When I think about the past, I remember only bad memories.**
9. Chance is a prerequisite to success, and only others have the chance to succeed.**

Responses were scored on a 4 point Likert scale: *strongly* *agree* = 4, *agree* = 3, *disagree* = 2, *strongly* *disagree* = 1.

** Items reverse scored
