# Supplementary material for: Development and Assessment of the Personal Emotional Capital Questionnaire for Adults
Source: Int J Environ Res Public Health. 2021 Feb 14;18(4):1856. doi: 10.3390/ijerph18041856 (PMC7918152; doi:10.3390/ijerph18041856)
Supplement: Supplementary file 1 [file ijerph-18-01856-s001.zip › ijerph-1066509-IR-SI-supplementary/New Questionnaire.docx]

| Please read each statement and rate how true each statement is for you: | Strongly agree | Agree | Disagree | Strongly  disagree |
| --- | --- | --- | --- | --- |
| 1. I learn from my mistakes and failures and from these I gain experience. |  |  |  |  |
| 1. I know my weaknesses and strengths. |  |  |  |  |
| 1. I am a highly-motivated person (if I decide to do something, I will do it). |  |  |  |  |
| 1. I can correct my mistakes and help myself progress in my job or learning and other affairs. |  |  |  |  |
| 1. I can set goals for myself. |  |  |  |  |
| 1. When I am aware of my mistakes and weaknesses or try to analyze them, I do not feel bad. |  |  |  |  |
| 1. I do not understand why I feel miserable (I do not know why I fail). |  |  |  |  |
| 1. When I get upset or angry, I do or say things that I will regret later. |  |  |  |  |
| 1. I am a valuable person. |  |  |  |  |
| 1. I am satisfied with my face and body (my looks). |  |  |  |  |
| 1. I am proud of and satisfied with myself. |  |  |  |  |
| 1. I can be as successful as other people. |  |  |  |  |
| 1. I usually successfully accomplish the work that assigned to me or that I voluntarily undertake. |  |  |  |  |
| 1. I love myself as I am. |  |  |  |  |
| 1. I can defend my rights without the help of others. |  |  |  |  |
| 1. I always know what to say to others. |  |  |  |  |
| 1. I feel humiliated (inferior to others and worthless). |  |  |  |  |
| 1. Sometimes I feel like a useless person. |  |  |  |  |
| 1. If I participate in election polls, my vote is not affected by my friends, colleagues or family. I decide to whom I vote for. |  |  |  |  |
| 1. I listen to others' opinions and advice, but I'm not dependent on others to make decisions. I make the final decision. |  |  |  |  |
| Please read each statement and rate how true each statement is for you: | Strongly agree | Agree | Disagree | Strongly  disagree |
| 1. I do what I think is right, no matter that others are against me, or what they say. |  |  |  |  |
| 1. When choosing clothes or hairstyles, I do not care about what others are wearing or what the latest fashion is. I choose clothes or hairstyles that I see fit and I like. |  |  |  |  |
| 1. I can say no to others (colleagues, friends, family, etc.) even if they oppose me or put pressure on me. |  |  |  |  |
| 1. I accept responsibility for my mistakes and failures. |  |  |  |  |
| 1. What others think about me is not important to me and does not affect my thoughts, ideas and plans. |  |  |  |  |
| 1. I do not feel the need to change my behavior or the way I talk, to satisfy others and gain their approval. |  |  |  |  |
| 1. I always agree with others’ opinions when it comes to movies, books, sports teams, and actors. I don’t have any strong personal opinions. |  |  |  |  |
| 1. When talking to others at home, university or work, I have no idea of myself and I accept whatever others say. |  |  |  |  |
| 1. If I do not understand something or have a question in the classroom or workplace, I will ask the professor or the person in charge. |  |  |  |  |
| 1. When I am with my friends, colleagues or family, I say my own opinion even if everyone is opposed to me. |  |  |  |  |
| 1. If I do something wrong, I have the courage to apologize. |  |  |  |  |
| 1. If someone (family members, professors, employers and colleagues, etc.) criticizes or says something unfair about me, I can talk to them and defend myself. |  |  |  |  |
| Please read each statement and rate how true each statement is for you: | Strongly agree | Agree | Disagree | Strongly  disagree |
| 1. I let others comment, even if they don’t agree with me. |  |  |  |  |
| 1. When I need help, I can tell others and ask for help. |  |  |  |  |
| 1. If after a purchase I find out that the seller has given me a defective or poor quality item, I can return the product. |  |  |  |  |
| 1. If others (friends, colleagues, family members, etc.) insist that I do something, I do it, even if I would rather do something else (it's hard for me to say no to others). |  |  |  |  |
| 1. When a discussion is over, I regret that I didn’t have enough courage to say my opinion. |  |  |  |  |
| 1. There is always a way to progress. |  |  |  |  |
| 1. Every day I can learn something new. |  |  |  |  |
| 1. I know what my goal is in life and what I am looking for. |  |  |  |  |
| 1. I can perform my duties well. |  |  |  |  |
| 1. I know what my talents are. |  |  |  |  |
| 1. I try to discover and develop my talents. |  |  |  |  |
| 1. My life is neat, organized and based on a plan. |  |  |  |  |
| 1. I try every day to improve my skills and abilities. |  |  |  |  |
| 1. I refrain from trying to solve complex problems, doing hard works and tackling crises. |  |  |  |  |
| 1. I feel exhausted and frustrated. |  |  |  |  |
| 1. I can talk to groups, colleagues or friends and encourage them to do something. |  |  |  |  |
| 1. I can guide groups, friends or colleagues by providing different examples. |  |  |  |  |
| 1. I do not interrupt others when they talking and I understand them well. |  |  |  |  |
| Please read each statement and rate how true each statement is for you: | Strongly agree | Agree | Disagree | Strongly  disagree |
| 1. If someone criticizes me, I do not get angry. I listen to them. |  |  |  |  |
| 1. I can mingle with the students from other fields of study at my university or with other people at a workplace and make friends with them. |  |  |  |  |
| 1. If there is a problem between myself and others, I can talk that problem over with them and solve it. |  |  |  |  |
| 1. In order to have good relationships with others, I pay attention to their feelings and attitudes. |  |  |  |  |
| 1. I have a close relationship with my friends. |  |  |  |  |
| 1. I cannot find a topic to talk about with others. |  |  |  |  |
| 1. I'm aloof and quiet among my classmates, colleagues, family members or relatives. |  |  |  |  |
| 1. Before I criticize someone, I put myself in their place, and I think if I were them, what I would do. |  |  |  |  |
| 1. I try to understand others' needs and help them. |  |  |  |  |
| 1. When my friends, colleagues or classmates at university have a problem, I sympathize with them. |  |  |  |  |
| 1. When I see someone being oppressed, I'd love to help them. |  |  |  |  |
| 1. When I see an angry or upset person, I try to talk to them and calm him down. |  |  |  |  |
| 1. When I become aware of my friends’, colleagues’ or classmates’ problems, I try to offer them different solutions. |  |  |  |  |
| 1. I respect other people’s opinions. |  |  |  |  |
| 1. When I see someone get hurt, I keep calm so I can help him. |  |  |  |  |
| 1. Hearing an opposing opinion makes me feel angry. |  |  |  |  |
| Please read each statement and rate how true each statement is for you: | Strongly agree | Agree | Disagree | Strongly  disagree |
| 1. The problems and needs of others do not concern me. |  |  |  |  |
| 1. When faced with a problem or difficult situation, I first gather information about it and consult with others. |  |  |  |  |
| 1. When I try to solve a problem or manage a crisis, I first consider all the available options and then I come up with the best solution. |  |  |  |  |
| 1. I can change my old habits. |  |  |  |  |
| 1. If I do not reach a goal, I can define new goals for myself. |  |  |  |  |
| 1. I can manage and plan household chores, college work, and tasks that should be done at my workplace. |  |  |  |  |
| 1. There are several solutions to each problem or crisis. |  |  |  |  |
| 1. When I fail, it's not hard for me to start again. |  |  |  |  |
| 1. It is not hard for me to make a change in life (form good, new habits). |  |  |  |  |
| 1. When faced with a problem or difficult situation, it is hard for me to decide on the appropriate solution. |  |  |  |  |
| 1. I can control my feelings (I do not cry, I do not scream and shout, etc.). |  |  |  |  |
| 1. During failures, I remain hopeful and cool and I do not get disappointed. |  |  |  |  |
| 1. Even if I am in a bad situation, I can think straight and I do not lose control. |  |  |  |  |
| 1. I know how to keep calm under difficult conditions. |  |  |  |  |
| 1. When I know that if I do or say something, it will make things worse, I keep silent and don’t do anything. |  |  |  |  |
| 1. I have no problem concentrating while studying and learning. |  |  |  |  |
| 1. It is difficult for me to quit bad habits. |  |  |  |  |
| Please read each statement and rate how true each statement is for you: | Strongly agree | Agree | Disagree | Strongly  disagree |
| 1. Every morning I am happy and cheerful. |  |  |  |  |
| 1. I accept “where there is a will, there is way”. |  |  |  |  |
| 1. People are inherently good and can be trusted. |  |  |  |  |
| 1. I know that under the most difficult circumstances I can work to reach my goals. |  |  |  |  |
| 1. Before I start a task, I feel that I can pull it off. |  |  |  |  |
| 1. I always expect that problems and hardships will to lead to a happy ending. (of course this is not the case all the time). |  |  |  |  |
| 1. I speak more about the future than the past. |  |  |  |  |
| 1. When I think about the past, I remember only bad memories. |  |  |  |  |
| 1. Chance is a prerequisite to success, and only others have the chance to succeed. |  |  |  |  |
